# A Novel Approach to Determining Violence Risk in Schizophrenia: Developing a Stepped Strategy in 13,806 Discharged Patients

**DOI:** 10.1371/journal.pone.0031727

**Published:** 2012-02-16

**Authors:** Jay P. Singh, Martin Grann, Paul Lichtenstein, Niklas Långström, Seena Fazel

**Affiliations:** 1 Department of Psychiatry, University of Oxford, Warneford Hospital, Oxford, United Kingdom; 2 Centre for Violence Prevention, Karolinska Institutet, Stockholm, Sweden; 3 Department of Medical Epidemiology and Biostatistics, Karolinska Institutet, Stockholm, Sweden; Institute of Psychiatry at the Federal University of Rio de Janeiro, Brazil

## Abstract

Clinical guidelines recommend that violence risk be assessed in schizophrenia. Current approaches are resource-intensive as they employ detailed clinical assessments of dangerousness for most patients. An alternative approach would be to first screen out patients at very low risk of future violence prior to more costly and time-consuming assessments. In order to implement such a stepped strategy, we developed a simple tool to screen out individuals with schizophrenia at very low risk of violent offending. We merged high quality Swedish national registers containing information on psychiatric diagnoses, socio-demographic factors, and violent crime. A cohort of 13,806 individuals with hospital discharge diagnoses of schizophrenia was identified and followed for up to 33 years for violent crime. Cox regression was used to determine risk factors for violent crime and construct the screening tool, the predictive validity of which was measured using four outcome statistics. The instrument was calibrated on 6,903 participants and cross-validated using three independent replication samples of 2,301 participants each. Regression analyses resulted in a tool composed of five items: male sex, previous criminal conviction, young age at assessment, comorbid alcohol abuse, and comorbid drug abuse. At 5 years after discharge, the instrument had a negative predictive value of 0.99 (95% CI = 0.98–0.99), meaning that very few individuals who the tool screened out (n = 2,359 out of original sample of 6,903) were subsequently convicted of a violent offence. Screening out patients who are at very low risk of violence prior to more detailed clinical assessment may assist the risk assessment process in schizophrenia.

## Introduction

Although schizophrenia is consistently associated with an increased risk of violence compared with general population controls in different countries and using various definitions of violence, most individuals with schizophrenia are not dangerous [1], [2]. A recent population study estimated rates of violent crime to be approximately 10–15% after diagnosis [1], and cohort studies have reported rates between 8–14% [3]–[6]. Despite this, current treatment guidelines in the US and UK recommend that violence risk be assessed for all individuals diagnosed with schizophrenia [7], [8], and over 100 instruments have been developed to assist in the prediction of such behaviour in general and psychiatric populations [9], [10]. Available instruments focus on identifying those patients with schizophrenia who are at highest risk of violence and typically require several hours to administer, placing a considerable burden on the resources of mental health services [11], [12].

One approach that could improve the risk assessment process is to develop instruments to screen out patients who are at very low risk of future violence that can be used prior to in-depth assessments of dangerousness (in contrast to current one-step methods that administer costly risk assessments to all patients without initial screening). This stepped approach is similar to that used by many diagnostic screening tools in physical medicine, such as the use of mammography or prostatic specific antigen screening for breast or prostate cancer, respectively, prior to biopsy [13], and allows for clinical resources, including more detailed clinical risk assessment, to be targeted at those more likely to need intervention. In addition, using such an approach would assist in risk management by identifying individuals who could be targeted for treatment in those who are not screened out.

The objective of the present study was to develop a screening tool to identify individuals with schizophrenia who are at very low risk of violence after hospital discharge. We developed and cross-validated the instrument using a cohort of 13,806 discharged patients with schizophrenia, with a potential follow-up of 33 years. Rather than simply turning risk assessment on its head (i.e., substituting factors such as previous criminal conviction with no previous conviction), the tool was statistically developed to maximise sensitivity (i.e. identifying all those individuals not at very low risk) for use as part of a stepped strategy. We aimed to create a tool composed of routinely available factors that was potentially scalable as it does not require specific training or additional costs. These properties have been identified as important for any instrument aiming to be clinically relevant [14].

## Methods

### Study protocol

The Standards for Reporting of Diagnostic Accuracy Studies (STARD) Statement [15], a 25-item checklist of reporting characteristics, was followed.

### Data registries

Data was collected from the following nationwide population-based registers in Sweden: The Hospital Discharge Register (HDR; held at the National Board of Health and Welfare), the Migration Register (Statistics Sweden), the Cause of Death Register (National Board of Health and Welfare), the National Crime Register (National Council for Crime Prevention), Education Registers (Statistics Sweden), and the Multi-Generation Register (Statistics Sweden). Merging these datasets was possible as all national registers use residents' (including immigrants) same 10-digit personal identification numbers. An independent government organisation (Statistics Sweden) merged the registers and, upon assigning each participant a novel case number, destroyed the coding sheet that linked case numbers and personal identification numbers. This anonymisation made informed consent unnecessary. The Ethics Committee at the Karolinska Institutet approved this protocol (2005/174/31/4).

### Participants

Using the HDR, all individuals aged 15 years (the age of criminal responsibility) and older who had been admitted to hospital for assessment and/or treatment in Sweden and had been discharged between January 1, 1973 and December 31, 2004 with a diagnosis of schizophrenia were identified. The HDR is a high quality register with less than 1% of hospital discharges missing personal identification numbers [16]. Diagnoses of schizophrenia were made using the *International Classification of Diseases* (ICD-8: 295; ICD-9: 295; ICD-10: F20). Only those individuals who had been diagnosed with schizophrenia on at least two occasions (*N* = 13,806) were included to improve specificity [17].

Participants were followed until their first conviction or until they were censored due to emigration (data obtained from the Migration Register), death (data obtained from the Cause of Death Register), or the end of the follow-up period (December 31, 2004).

### Calibration and cross-validation samples

The population cohort was randomly divided into two samples of 6,903 participants. One of the samples was used to develop the screening tool and the other to cross-validate it. As the use of regression to develop risk instruments has been criticised for exaggerating predictive validity estimates in calibration samples [18], [19], the cross-validation sample was randomly split into three independent subsamples, each consisting of 2,301 participants. Cross-validating the tool using these subsamples provided the opportunity to assess shrinkage effects.

### Definition of violence

Violent conviction data was extracted from the National Crime Register for the years 1973–2004. The National Crime Register is a high quality register, missing only 0.05% of personal identification numbers between 1988–2000 [16]. Violent crime was defined as having received a conviction for homicide, assault, robbery, arson, any sexual offence, illegal threats, or intimidation. The inclusion of noncontact offences in the operational definition of violence is consistent with previous risk assessment research [20], [21], including studies that have specifically investigated the association between schizophrenia and criminal behaviour [1]. Nonviolent offences such as fraud and perjury were not included, as they were perceived to be of less clinical interest than more serious behavioural outcomes and as they are not referenced in current treatment guidelines for schizophrenia [7], [8]. Participants were coded as either having or not having a conviction for a violent crime during follow-up. Conviction data accurately reflects resolved criminality in this population as plea-bargaining is not permitted in Sweden and as individuals are convicted as guilty regardless of mental illness.

### Outcome measures

Four outcome statistics were used to measure predictive validity: negative predictive value (NPV), positive predictive value (PPV), diagnostic odds ratio (DOR), and area under the curve (AUC). These statistics are commonly used outcome measures in the medical diagnostic literature [22].

The NPV is the proportion of individuals who are predicted by a tool not to offend who do not offend, whereas the PPV represents the proportion of individuals who are predicted to offend who actually offend. Both statistics are base rate dependent. As the ability to accurately classify individuals as being at risk of violence in psychiatric populations is highly dependent upon base rate [23], this may be considered a strength.

The DOR is the ratio of the odds of a true positive relative to the odds of a false positive. In addition to being independent of the base rate of offending, the DOR is also useful in that researchers and clinicians are familiar with the concept of an odds ratio, making the statistic easier to comprehend for non-specialists [24].

The receiver operating characteristic (ROC) curve plots an instrument's true positive rate against its false positive rate across score thresholds. The area under this curve (AUC) is the probability that a randomly selected offender has a higher test score than a randomly selected non-offender [25]. The statistic can be considered an index of how well a tool discriminates between offenders and non-offenders. As with the DOR, the AUC is not base rate dependent.

### Risk factors

We prioritised the inclusion of risk factors that could be scored using routinely collected and accessible file information. These variables included male sex [26], [27], previous criminal conviction [27], age at assessment [27], comorbid alcohol abuse [28], and comorbid drug (non-alcohol) abuse [28].

Information on sex was extracted from the HDR. Previous criminal conviction referred to any crime (violent or not) before hospital discharge, and was gathered from the National Crime Register. The age at which participants were discharged from hospital with a second diagnosis of schizophrenia ranged from 15 to 54 years (*Median* = 26; *Interquartile Range* = 22–33). We used the Chi-squared Automatic Interaction Detector (CHAID) method adjusted by time at risk to identify the age cut-off which best classified participants as being at risk of violent conviction [29]. Data on participants' hospital admissions during the years 1973–2004 were extracted from the HDR and examined for evidence of principal or comorbid diagnoses of alcohol abuse (ICD-8: 303; ICD-9: 303, 305.1; ICD-10: F10, except ×.5) and drug abuse (ICD-8: 304; ICD-9: 304, 305.9; ICD-10: F11-F19, except ×.5). This information was used as a marker of substance abuse comorbidity. A sensitivity analysis was also conducted for the following illicit drug categories: opiate, sedative, stimulant, hallucinogen, and polysubstance abuse.

Secondary risk factors tested included low level of education [30], having a parent who was convicted of a violent offence [31], and having a parent who was diagnosed with alcohol abuse [32]. These were considered secondary, as such information may not be routinely available. One study estimated that school records were utilized in approximately 15% of clinicians conducting risk assessments in adults [33]. Participants' highest level of completed education was collated from the Education Registers and a binary comparison was made between those who had completed compulsory school (a nine year comprehensive school for children aged 7 to 16) and those who had not.

We linked parents to the National Crime Register using the Multi-Generational Register to extract data on whether individuals had a father or mother who had previously been convicted of a violent crime. In addition, parents were linked to the HDR to extract data on diagnoses of alcohol abuse.

### Developing the screening tool

Analyses took place in two stages: in the first stage, the routinely accessible variables were combined into a five-item unit scored model (i.e., a model where the presence of a risk factor is scored as +1 and the absence as +0) and regressed with violent conviction as the dependent variable using Cox's proportional-hazards method [34]. The rates of true positives (TP), false positives (FP), true negatives (TN), and false negatives (FN) were calculated for the resulting tool at each risk score at 1, 2, and 5 years follow-up. Participants who were not at risk for 1, 2, or 5 years (respectively) due to emigration, death, or end of follow-up were excluded from these calculations.

When selecting a cut-off score, the relative costs of false negatives and false positives were taken into consideration. The societal and political costs associated with screening out a patient who would go on to commit a violent crime were considered to outweigh the costs associated with conducting more detailed risk assessments for patients who would not go on to commit a violent offence. Therefore, the ideal cut-off point was identified as the risk score that maximised sensitivity while balancing specificity. Using this cut-off, the instrument's NPV, PPV, and DOR were calculated at each length of follow-up, as was AUC using receiver operating characteristic analysis.

In the second stage, each secondary variable was regressed with violent conviction as the dependent variable and ranked in order of their resulting hazard ratios. Variables were added one at a time to the primary model and, provided the added factor independently accounted for variation in the dependent variable, the revised tool's predictive validity was assessed at 1, 2, and 5 years follow-up. Effect estimates were compared with those produced by the five-item model at the same length of follow-up using 95% confidence intervals (CI). Additional comparisons were made using the *χ^2^* test of differences between proportions, Breslow and Day's *χ^2^* test of homogeneity between odds ratios [35], and Hanley and McNeil's *z* test for differences in AUC [36]. This process was repeated for each additive iteration.

### Cross-validation

The version of the screening tool that produced the highest rates of predictive validity using the fewest number of items was then cross-validated using the three replication subsamples. The effect estimates produced by the tool in each independent subsample at 1, 2, and 5 years follow-up were compared with those produced by the calibration sample at the same length of follow-up to examine evidence of shrinkage.

Analyses were conducted using SPSS 17.0.1 for Windows [37], STATA/IC 10.1 for Windows [38], and MedCalc 11.3.8.0 for Windows [39].

## Results

### Description of the samples

The population cohort consisted of 13,806 individuals with two or more hospital discharge diagnoses of schizophrenia during January 1, 1973 and December 31, 2004 (Table 1). The cohort was randomly divided into a calibration sample (*n* = 6,903) and three independent cross-validation subsamples (*n* = 2,301 for each).

**Table 1:** Descriptive characteristics of the calibration and cross-validation samples of a violence screening tool for individuals with hospital discharge diagnoses of schizophrenia.

		Sample				
Domain	Variable	Calibration sample (*n* = 6903)	Cross-validation sample 1 (*n* = 2301)	Cross-validation sample 2 (*n* = 2301)	Cross-validation sample 3 (*n* = 2301)	Adjusted HR^a^(95% CI)
Demographic factors	Male sex, *n* (%)	4392 (63.6)	1480 (64.3)	1504 (65.4)	1515 (65.8)	3.3 (2.7–4.0)
	Age at assessment (in years), mean (SD)	28.9 (7.2)	28.9 (7.2)	29.2 (7.2)	29.2 (7.3)	1.9 (1.6–2.3)
	Non-completion of compulsory school, *n* (%)	2005 (29.1)	660 (28.7)	679 (29.5)	692 (30.1)	1.4 (1.2–1.6)
Individual factors	Previous criminal conviction, *n* (%)	2806 (40.7)	899 (39.1)	974 (42.3)	978 (42.5)	3.3 (2.8–3.8)
	Alcohol abuse comorbidity, *n* (%)	1078 (15.6)	353 (15.3)	353 (15.3)	331 (14.4)	2.9 (2.5–3.4)
	Drug (non-alcohol) abuse comorbidity, *n* (%)	1151 (16.7)	360 (15.6)	389 (16.9)	357 (15.5)	3.5 (3.1–4.0)
Familial factors	Father convicted of a violent offence, *n* (%)	127 (1.8)	41 (1.8)	39 (1.7)	35 (1.5)	2.3 (1.7–3.2)
	Mother convicted of a violent offence, *n* (%)	17 (0.2)	7 (0.3)	6 (0.3)	7 (0.3)	2.8 (1.4–5.3)
	Father alcohol abuse comorbidity, *n* (%)	539 (7.8)	188 (8.2)	163 (7.1)	147 (6.4)	1.4 (1.1–1.8)
	Mother alcohol abuse comorbidity, *n* (%)	179 (2.6)	56 (2.4)	60 (2.6)	68 (3.0)	1.8 (1.3–2.5)
Base rate of violent conviction^b^	*n* (%)	887 (12.9)	309 (13.4)	321 (14.0)	275 (12.0)	

**Note:** HR = hazard ratio; CI = confidence interval; SD = standard deviation.

a Adjusted by age at assessment and sex using participants from the calibration sample.

b Between January 1, 1973 and December 31, 2004.

### Calibrating the screening tool

Using the CHAID method, we identified 32 years as the age cut-off that best classified participants as being at risk of violent conviction for the “young age at assessment” variable.

Cox regression found that the five routinely accessible factors (i.e., male sex, previous criminal conviction, young age at assessment, comorbid alcohol abuse, and comorbid drug abuse) were significant independent predictors of the incidence of violent conviction (Table 1).

The TP, FP, TN, and FN rates were calculated for the five-item tool at each risk score at 1, 2, and 5 years follow-up. The optimal cut-off score was identified as +2 (Table 2). Of the 2,359 participants who scored below this threshold, 2,353 were not convicted of a violent offence within 1 year after hospital discharge (NPV = 0.99; 95% CI = 0.99–1.00). The screening tool's PPV at 1 year after discharge was 0.01 (95% CI = 0.01–0.02), the DOR was 3.79 (95% CI = 1.65–8.72), and the AUC was 0.67 (95% CI = 0.66–0.68). There was a trend towards PPVs, DORs, and AUCs increasing over 2 and 5 years follow-up, while NPVs remained at 99% (Table 3). To investigate the instrument's performance with different base rates, we measured PPVs and NPVs at 1, 2, and 5 years follow-up at base rates of 0%, 2%, 4%, 6%, 8%, and 10% (Table 4). While PPVs increased markedly with increasing base rates, NPVs remained above 95%.

**Table 2:** Rates of true positives, true negatives, false positives, and false negatives by risk score for the calibration sample of a violence screening tool for individuals with hospital discharge diagnoses of schizophrenia.

Length of follow-up	Risk score	True positives	True negatives	False positives	False negatives	Sensitivity	Specificity
1 year (*n* = 6645)	0	47	0	6598	0	1.00	0.00
	1	46	604	5994	1	0.98	0.09
	2*	41	2353	4245	6	0.87	0.36
	3	26	4493	2105	21	0.55	0.68
	4	14	5750	848	33	0.30	0.87
	5	3	6380	218	44	0.06	0.97
2 years (*n* = 6407)	0	93	0	6314	0	1.00	0.00
	1	90	573	5741	3	0.97	0.09
	2*	83	2245	4069	10	0.89	0.36
	3	58	4300	2014	35	0.62	0.68
	4	29	5505	809	64	0.31	0.87
	5	9	6104	210	84	0.10	0.97
5 years (*n* = 5666)	0	224	0	5442	0	1.00	0.00
	1	216	475	4967	8	0.96	0.09
	2*	202	1961	3481	22	0.90	0.36
	3	138	3762	1680	86	0.62	0.69
	4	64	4776	666	160	0.29	0.88
	5	15	5261	181	209	0.07	0.97

**Note:** Participants from the calibration sample were excluded if they were not at risk for 1, 2, or 5 years (respectively).

*Cut-off score.

**Table 3:** Comparison of outcome measures calculated during calibration and cross-validation of a violence screening tool for individuals with hospital discharge diagnoses of schizophrenia.

		Outcome measure			
Length of follow-up	Sample	NPV (95% CI)	PPV (95% CI)	DOR (95% CI)	AUC (95% CI)
1 year	Calibration sample (*n* = 6645)	0.99 (0.99–1.00)	0.01 (0.01–0.02)	3.79 (1.65–8.72)	0.67 (0.59–0.74)
	Cross-validation sample 1 (*n* = 2205)	0.99 (0.99–1.00)	0.01 (0.01–0.02)	5.16 (1.33–20.04)	0.74 (0.62–0.85)
	Cross-validation sample 2 (*n* = 2205)	0.99 (0.99–1.00)	0.01 (0.01–0.02)	2.07 (0.73–5.93)	0.66 (0.54–0.78)
	Cross-validation sample 3 (*n* = 2190)	0.99 (0.99–1.00)	0.01 (0.01–0.02)	5.14 (1.34–19.93)	0.62 (0.53–0.71)
2 years	Calibration sample (*n* = 6407)	0.99 (0.99–1.00)	0.02 (0.02–0.03)	4.58 (2.40–8.74)	0.69 (0.64–0.74)
	Cross-validation sample 1 (*n* = 2136)	0.99 (0.99–1.00)	0.02 (0.02–0.03)	17.89 (3.08–103.80)	0.75 (0.67–0.82)
	Cross-validation sample 2 (*n* = 2111)	0.99 (0.98–1.00)	0.02 (0.02–0.03)	2.56 (1.09–6.03)	0.67 (0.58–0.75)
	Cross-validation sample 3 (*n* = 2111)	0.99 (0.99–1.00)	0.02 (0.02–0.03)	3.12 (1.24–7.82)	0.65 (0.57–0.73)
5 years	Calibration sample (*n* = 5666)	0.99 (0.98–0.99)	0.06 (0.05–0.06)	5.17 (3.33–8.03)	0.69 (0.66–0.73)
	Cross-validation sample 1 (*n* = 1875)	0.99 (0.98–0.99)	0.06 (0.05–0.06)	4.10 (2.12–7.91)	0.68 (0.63–0.74)
	Cross-validation sample 2 (*n* = 1879)	0.98 (0.97–0.99)	0.05 (0.05–0.06)	3.08 (1.63–5.81)	0.68 (0.62–0.74)
	Cross-validation sample 3 (*n* = 1868)	0.98 (0.97–0.99)	0.05 (0.04–0.05)	2.21 (1.23–3.95)	0.66 (0.60–0.72)

**Note:** NPV = negative predictive value; PPV = positive predictive value; DOR = diagnostic odds ratio; AUC = area under the curve; CI = confidence interval. Participants from the calibration and cross-validation samples were excluded if they were not at risk for 1, 2, or 5 years (respectively). All AUCs were significantly higher than chance. Test statistics reported no significant differences between the effect estimates produced by the calibration and cross-validation samples. The instrument's PPV increased significantly over time in both the calibration and the cross-validation samples. Differences in DOR and AUC were non-significant despite evidence of non-overlapping confidence intervals.

**Table 4:** A comparison of the positive and negative predictive values for a violence screening tool for individuals with hospital discharge diagnoses of schizophrenia across different base rates of violent conviction.

		Base rate of violent conviction					
Length of follow-up	Outcome measure	0%	2%	4%	6%	8%	10%
1 year (*n* = 6645)	NPV (95% CI)	0.99 (0.99–1.00)	0.99 (0.99–1.00)	0.99 (0.98–0.99)	0.98 (0.97–0.98)	0.97 (0.97–0.98)	0.96 (0.96–0.97)
	PPV (95% CI)	0.01 (0.00–0.01)	0.03 (0.01–0.07)	0.05 (0.03–0.08)	0.08 (0.06–0.11)	0.11 (0.08–0.13)	0.13 (0.11–0.15)
2 years (*n* = 6407)	NPV (95% CI)	0.99 (0.99–1.00)	0.99 (0.99–1.00)	0.99 (0.98–0.99)	0.98 (0.97–0.98)	0.97 (0.97–0.98)	0.97 (0.96–0.97)
	PPV (95% CI)	0.01 (0.00–0.01)	0.03 (0.01–0.07)	0.05 (0.03–0.09)	0.08 (0.06–0.11)	0.11 (0.09–0.13)	0.13 (0.11–0.16)
5 years (*n* = 5666)	NPV (95% CI)	0.99 (0.99–1.00)	0.99 (0.99–1.00)	0.99 (0.98–0.99)	0.98 (0.97–0.98)	0.98 (0.97–0.98)	0.97 (0.96–0.97)
	PPV (95% CI)	0.01 (0.00–0.01)	0.03 (0.01–0.07)	0.06 (0.04–0.09)	0.08 (0.06–0.11)	0.11 (0.09–0.13)	0.14 (0.11–0.16)

**Note:** PPV = positive predictive value; NPV = negative predictive value; CI = confidence interval. Values based on participants from the calibration sample. Participants were excluded if they were not at risk for 1, 2, or 5 years (respectively).

Univariate analyses also found that a number of secondary variables were significantly associated with the incidence of violent conviction (Table 1). However, we found no evidence of increased predictive validity when low level of education, parental conviction for a violent offence, or parental alcohol abuse comorbidity were added as items (results not shown). In addition, in a sensitivity analysis, no evidence of incremental predictive validity was found if one of opiate, sedative, stimulant, hallucinogen, or polysubstance abuse was included on the simple screening tool instead of any drug abuse.

### Cross-validation

Statistical tests revealed no significant differences between the effect estimates produced by the five-item instrument in the calibration and cross-validation samples at each length of follow-up, suggesting no clear shrinkage effects (Table 3). Of the 2,288 participants in the replication samples who were classified as low risk, 2,280 did not go on to be convicted of a violent offence within 1 year of hospital discharge (NPV = 0.99; 95% CI = 0.99–1.00). Similar to the calibration sample, there was a trend towards PPVs, DORs, and AUCs increasing over time, while NPVs remained at or above 98% at 2 and 5 years of follow-up (Table 3).

## Discussion

Using data collected from Swedish national registers over three decades, we investigated violence risk in 13,806 patients with schizophrenia in order to develop a simple screening tool that could accurately identify individuals who would not be convicted of a violent offence after hospital discharge. The instrument was composed of five routinely available risk factors: male sex, previous criminal conviction, young age at assessment, comorbid alcohol abuse, and comorbid drug abuse. We found that the tool could be accurately used to make “rule out” decisions (i.e., identifying who will not go on to violently offend), suggesting potential utility as a screening tool that could be used as part of a stepped approach to risk assessment. In this approach, very low risk patients are screened out, allowing for more detailed and resource-intensive assessment and management on the remaining (or screen positive) patients. The reported tool requires no training to use and hence is potentially scalable.

### Inclusion of clinical override

**Figure 1:**
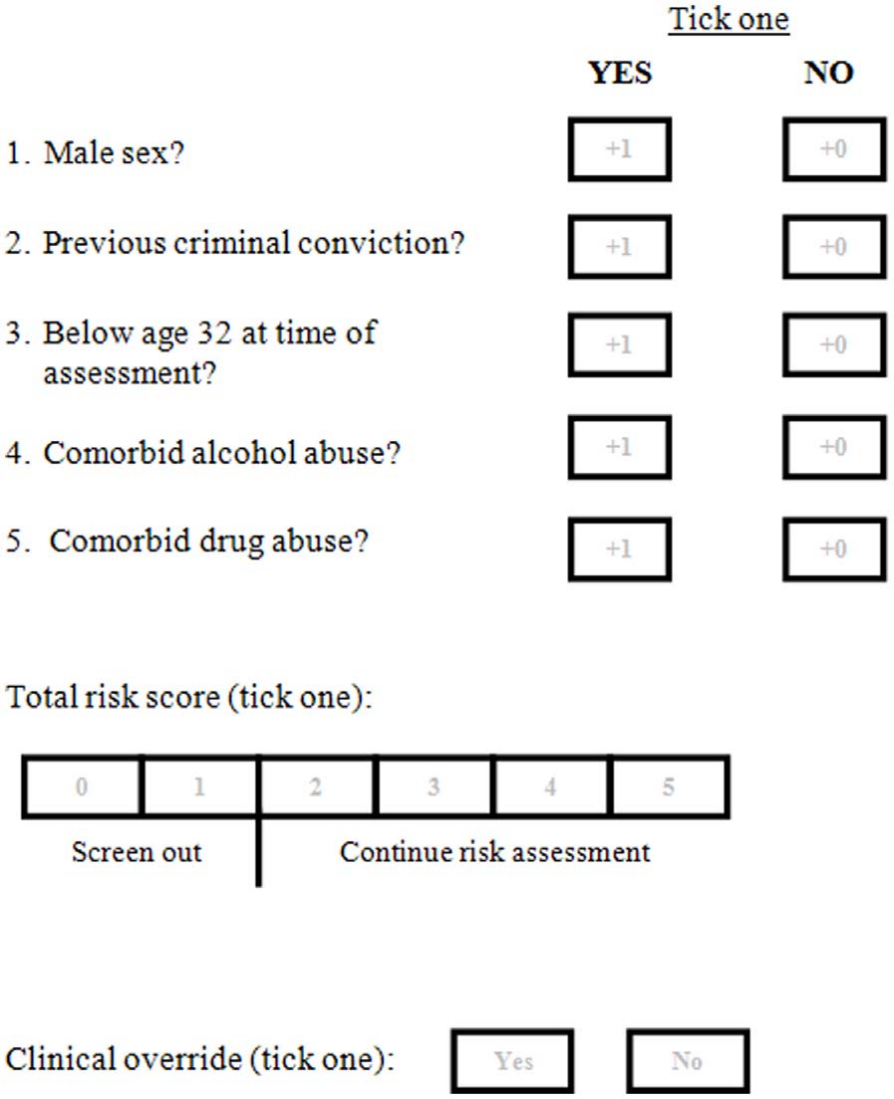
Proposed violence screening tool for individuals with schizophrenia.

The screening tool should complement the role of clinical judgement in violence risk assessment by identifying patients for whom detailed assessment may not be needed. Nevertheless, a number of risk factors that this tool does not measure may have clinical significance and more research is warranted to determine their value. For example, associations have been found between violence and non-adherence to medication [40], lack of insight [41], and psychotic symptoms such as persecutory delusions and command hallucinations [42]. In certain cases, clinicians may consider such factors as highly relevant (e.g., if they were associated with previous violence). Therefore, we have included a clinical override option in our coding sheet (Figure 1).

### Comparison with previous research

While several violence risk assessment instruments have been developed for general psychiatric populations [43]–[45], no tools to our knowledge have been designed as part of a stepped strategy for individuals with a specific psychiatric diagnosis [10]. In relation to the latter, as epidemiological investigations have shown that the base rate of violence varies by diagnosis [46] and existing tools may perform differently across diagnostic groups [10], general screening approaches are likely to lose sensitivity and specificity. Some evidence suggests that developing risk assessment tools for more specific populations may improve predictive validity [47]. One such instrument is an actuarial tool designed to predict assault in individuals diagnosed with psychotic disorders [48]. The calibration study for this instrument, a two-year clinical trial examining different models of community care in UK inner cities, found that demographic information was helpful in risk prediction [48], [49]. However. unlike the screening tool developed in the present study, this measure was designed to identify high risk individuals and hence produced a higher rate of false negatives (13% at 2 year follow-up compared with less than 1% in the present study).

The current screening instrument's success in screening out very low risk patients may be attributed to two sources. First, the tool was statistically developed so that it only includes risk factors that account for variance independently, and the selected cut-off maximises sensitivity and minimises the number of false negative predictions. Second, each of the risk factors has been proposed to lie on the causal pathway to violence. For example, increased levels of testosterone may predispose male patients with schizophrenia to aggression [50]. Further, there is evidence to suggest that the presence of the Met allele in the catechol-*O*-methyltransferase (COMT) Val158Met polymorphism, which has recently been put forth as an important neurobiological risk factor for violence in schizophrenia [51], increases violence risk solely in men [52]. Young age may increase violence risk, as aging reduces testosterone levels in both sexes [53] and is associated with improved emotion regulation [54]. Criminal history and diagnoses of alcohol or drug abuse may be markers of violence risk because they may reflect an antisocial lifestyle with an increased likelihood of having antisocial peers, procriminal attitudes, unemployment, exposure to destabilizers, and lack of adherence to treatment [55]. In addition, alcohol or drug use at the time of the violent offence may have acted as a disinhibitor. Despite this, this study was not designed to test causal mechanisms, and the screening tool clearly does not represent a causal model of violence risk.

To our knowledge, the simple screen presented in this report is the first risk assessment tool to have been designed to predict the likelihood of community violence in a specific diagnostic group, to have been constructed for use in identifying low as opposed to high risk patients, and to have been developed using a register-based sample of several thousand individuals, allowing for the calculation of more precise effect estimates than previous work. The calibration and cross-validation samples for the screening tool were several orders of magnitude larger than development studies for currently available actuarial risk assessment tools [56], [57].

### Implications

When used as part of a stepped approach to violence risk assessment, screen out tools can potentially save psychiatric services considerable resources although future research will have to test this in practice. Recent UK and US surveys have found that instruments which employ structured clinical judgement, where clinicians use empirically-based risk and protective factors to guide their predictions as to whether an individual will be violent, are amongst the most commonly used tools [58], [59]. With the time involved in administering such tools (familiarising oneself with the patient's case, collecting information from multiple sources to score items, conducting interviews, scoring the tool, and making a clinical judgement regarding risk level) and the costs involved (attending training sessions, purchasing tool manuals, and paying for each coding sheet), violence risk assessment, as often currently conducted, costs mental health services significant amounts of time and money. In a recent survey of forensic mental health professionals, Viljoen and colleagues [33] reported that using a structured tool to conduct a risk assessment takes an average of 15 hours and costs service providers an average of $100 USD per hour to complete. Therefore, screening tools that require little training, that are based on items scored using routinely available information, and can reduce caseload are potentially attractive. It should be noted, however, that patients with schizophrenia who are screened out will continue to need mental health resources to treat their illness. Therefore, while screening tools such as that developed in the present study may be of use in identifying patients who do not need management of their violence risk, they should not be used to ration treatment.

The results of the present investigation also suggest that reporting a single effect size does not provide an adequate picture of a risk assessment tool's predictive validity. Global effect estimates such as the AUC and the DOR do not measure the relative utility of an instrument in making “rule in” decisions (i.e., identifying who will go on to violently offend) versus “rule out” decisions (i.e., identifying who will not go on to violently offend). Had only AUCs or DORs have been reported for the simple tool in this study, different conclusions could have emerged about how the tool performed. AUCs below 0.70 may have been interpreted as evidence that the screening tool lacked any utility as a violence risk assessment instrument [60], whereas DORs above 5 may have been interpreted as evidence that the screen performed well in identifying both high and low risk patients [24]. It was only by examining the PPVs and NPVs of the tool that a clear picture emerged of how the instrument performed. Future studies investigating the predictive validity of forensic risk assessment tools should consider reporting a global effect estimate (e.g., AUC or DOR) as well as at least one “rule in” and “rule out” effect size (e.g., PPV and NPV, respectively).

### Limitations

There are several limitations of the present investigation. First, we relied on hospital data to identify schizophrenia cases. Therefore, our screen is applicable only to patients discharged from hospital, although other research has estimated that a minority of individuals in Sweden who have schizophrenia would not have been hospitalised over a 30-year period [61]. By excluding individuals with only a single discharge diagnosis, we will not have included all hospitalised individuals with schizophrenia. However, by including only those individuals with two diagnoses of schizophrenia, our sample had the advantage of diagnostic specificity [17]. That is, participants were less likely to have other diagnoses such as drug-induced psychosis or bipolar disorder. Related work has shown similar base rates of violence in those with one or more diagnoses of schizophrenia [1], so it is possible that the screen is generalisable to all hospitalised patients with the disorder. Further, as the prevalence [62], [63] and disability-adjusted life years [64] of individuals with schizophrenia and the rate of violent crime [65] in Sweden are similar to Western Europe and other regions, our findings may have some generalisability to other nations.

A second limitation was that the positive predictive values produced by the screening instrument were low. That is, patients who were not screened out by the simple tool were rarely convicted of violent crimes (i.e., false positives). This is likely a consequence of the low base rate of violence in individuals with schizophrenia and supports the assumption of the stepped approach that individuals who score highly on our tool should not be considered as moderate or high risk of violence, but rather as warranting a more comprehensive risk assessment. In this context, false positive predictions are not problematic, as a low-risk patient who is not screened out would receive what is currently treatment-as-usual: an in-depth clinically-based risk assessment. These findings highlight the challenge of identifying individuals at high risk of violence in populations with low base rates [66], and also the need for more research on improving structured risk instruments, possibly using other variables that we were unable to test using register-based measures.

A third limitation of the present investigation was that evidence of minimal shrinkage effects may have been due to our calibration and cross-validation samples having been selected from the same population, which had a low overall base rate of violence. Also, variables were coded the same way for both the construction and replication samples. Future work will have to test the predictive validity of the screening tool in different settings (e.g., non-hospitalised patients) and outcomes (e.g., violence not reported to the police).

A fourth limitation is that the screening tool does not discriminate in young men, who would all screen positive. Therefore, it may be that the screen provides the most clinical utility in women and older men. Future research could consider more discriminating tools in young men.

### Future Directions

Before the stepped approach to risk assessment could be considered in clinical practice, it would need further validation, including clinical studies in other countries to assess the generalisability of the present study's findings. These studies should be prospective and include both economic analyses (i.e., comparisons of resources consumed prior to implementation and after) and qualitative analyses to explore clinicians' perceptions of this novel approach.

In addition, as clinicians may be interested in tools that predict more than one adverse outcome [67], future studies may wish to investigate the utility of the screening instrument in the prediction of other adverse outcomes such as suicide. In support, a systematic review has found male sex, criminal history, young age, and substance abuse to be significant risk factors for suicide in schizophrenia [68]–[70].

To what extent the five factors included on the screening tool are specific to schizophrenia also needs further investigation. For two factors, it is unlikely: the age cut-off in this instrument (32 years) is older than the median age of offending in general populations [71], and rates of substance abuse comorbidity are substantially higher in patients with schizophrenia than general population samples [72].

### Conclusion

The aim of the present study was to develop a simple instrument to identify those individuals diagnosed with schizophrenia who are at very low risk of violence after hospital discharge. We aimed to design a tool that used routinely available information and was straightforward to administer. With reasonably high rates of accuracy, the use of five items, no complicated weighting algorithm, and an easy to interpret classification system (screen out vs. continue risk assessment), our findings suggest that using a stepped strategy in which very low risk patients are screened out prior to in-depth risk assessment may assist in improving the quality of violence risk assessment in schizophrenia.
